# A Human Sensory Pathway Connecting the Foot to Ipsilateral Face That Partially Bypasses the Spinal Cord

**DOI:** 10.3389/fnins.2019.00519

**Published:** 2019-05-22

**Authors:** Morry Silberstein, Andrew K. Nunn, Peter D. Drummond, Dawn Wong Lit Wan, Janette Alexander, Melinda Millard, Mary P. Galea

**Affiliations:** ^1^School of Molecular and Life Sciences, Curtin University, Perth, WA, Australia; ^2^Victorian Spinal Cord Service, Austin Health, Melbourne, VIC, Australia; ^3^School of Psychology, Murdoch University, Murdoch, WA, Australia; ^4^School of Health and Biomedical Sciences, RMIT University, Melbourne, VIC, Australia; ^5^Department of Medicine, University of Melbourne, Melbourne, VIC, Australia

**Keywords:** neuroanantomy, capsaicin, pain, C-fiber, afferent

## Abstract

Human sensory transmission from limbs to brain crosses and ascends through the spinal cord. Yet, descriptions exist of ipsilateral sensory transmission as well as transmission after spinal cord transection. To elucidate a novel ipsilateral cutaneous pathway, we measured facial perfusion following painfully-cold water foot immersion in 10 complete spinal cord-injured patients, 10 healthy humans before and after lower thigh capsaicin C-fiber cutaneous conduction blockade, and 10 warm-immersed healthy participants. As in healthy volunteers, ipsilateral facial perfusion in spinal cord injured patients increased significantly. Capsaicin resulted in contralateral increase in perfusion, but only following cold immersion and not in 2 spinal cord-injured patients who underwent capsaicin administration. Supported by skin biopsy results from a healthy participant, we speculate that the pathway involves peripheral C-fiber cross-talk, partially bypassing the cord. This might also explain referred itch and jogger's migraine and it is possible that it may be amenable to training spinal-injured patients to recognize lower limb sensory stimuli.

## Introduction

Transmission of afferent neural information in humans from the limbs to the brain requires an intact spinal cord (Millan, 1999). Propagation of lower limb pain sensations involves peripheral nociceptor activation, conduction via afferent nerves to the spinal cord and then, following synaptic connections, decussation, and transmission to the contralateral side of brain (Almeida et al., 2004). The neural pathways transmitting pain from the limbs to the brain in humans would be expected to be abolished following spinal cord transection. Yet, Villanueva et al. citing a personal communication from eminent British neurologist, the late Peter Nathan, suggested that noxious stimuli could still activate higher centers following surgical cordotomy, but the mechanism responsible could not be explained (Villanueva et al., 2006). There is also recent functional MRI evidence indicating that somatosensory neural transmission can bypass a complete spinal cord injury, but the exact pathway was not determined (Wrigley et al., 2018). Additionally, there are descriptions of sensory phenomena suggesting proximal propagation of afferent neural information in humans ipsilateral to the side of peripheral stimulation, such as extracranial vasodilatation following painful foot immersion (Drummond and Chung, 2012) and referred itch (Richter, 1977), but the nature of such pathways has yet to be elucidated. We speculated that a common neural pathway responsible for 3 phenomena—ascending pain transmission following cordotomy and complete spinal cord injury, ipsilateral extracranial vasodilatation following painful foot immersion, and referred itch—is located in the skin, partially bypassing the spinal cord, in a manner similar to antidromic and orthodromic conduction in neurogenic inflammation, partially bypassing the spinal cord (Chahl, 1988). Here we sought to demonstrate the existence of this pathway in humans in a group of patients with spinal cord injury and in 2 groups of paired healthy controls before and after conduction blockade of small fiber afferents in ipsilateral thigh skin.

## Materials and Methods

### Format

#### Controlled Before-and-After Study

Sample size was determined as follows: based on preliminary observations, we estimated a (Cohen's d) large effect size of at least 1.0 with percentage of non-overlap of over 50%. For alpha of 0.05, with mean difference between groups of 1.0 effect and pooled standard deviation of 0.7 effect, a sample size of 8 per group results in 80% power. We then set a slightly higher sample size of 10 per group to allow for possible variation in standard deviations, with *post hoc* analysis confirming our preliminary power estimate.

### Ethics

This study was performed in strict accordance with the recommendations of the National Health and Medical Research Council statement on Ethical Conduct in Human Research. All procedures were approved by the Institutional Human Research Ethics Committees of Austin Health (Approval No. H2013/05045), Curtin University (Approval Nos. SMEC-60-09 and RDSE-26-15), and Murdoch University (Approval No. 2018/159). All participants gave written informed consent in accordance with the Declaration of Helsinki.

### Participants

**Figure 1:**
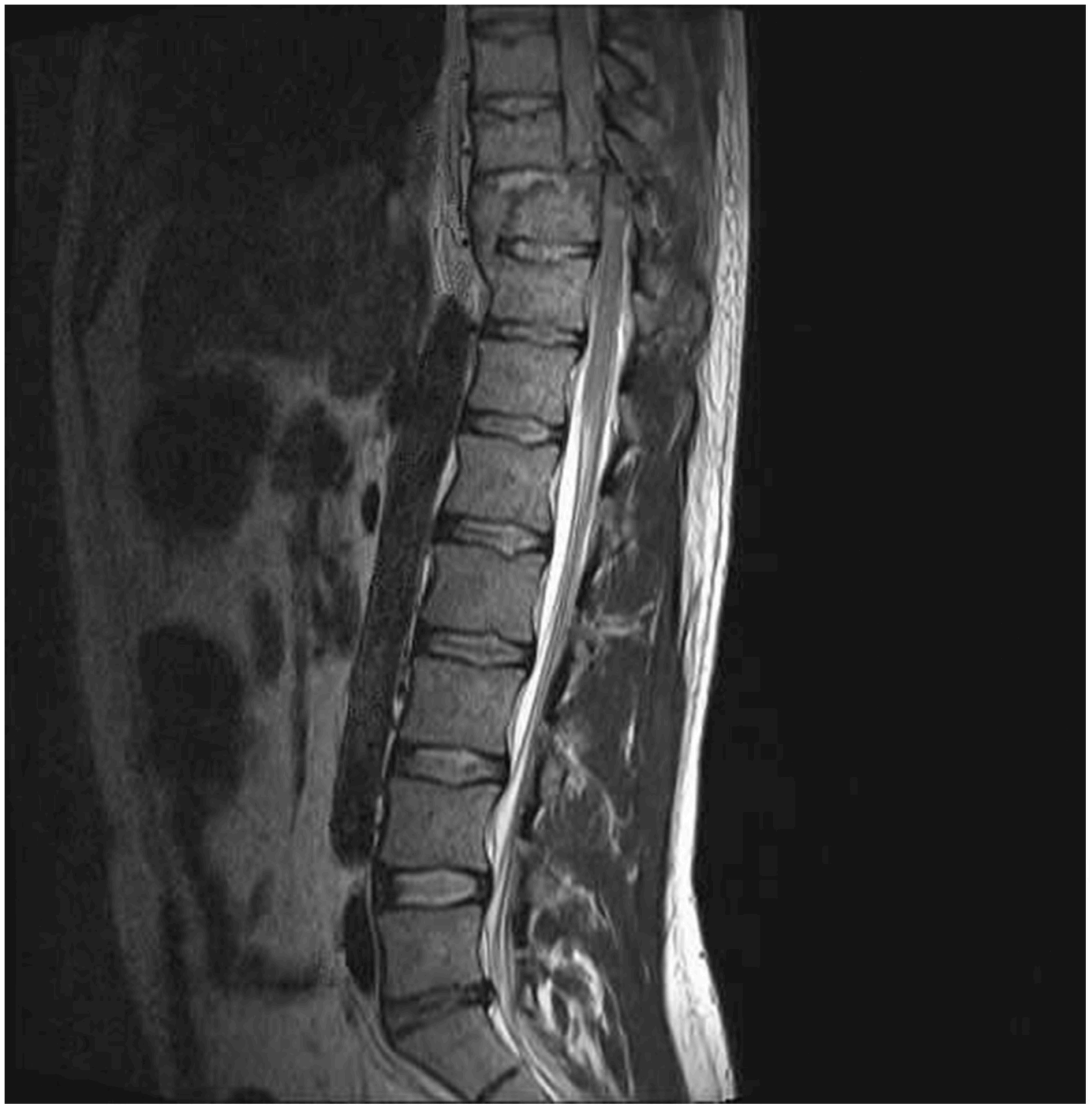
MRI sagittal image of included patient with complete spinal cord transection. All included spinal cord injured patients had MRI evidence of complete spinal cord transection. This image was obtained from a 25 year-old male with a T10 complete neurological deficit.

Spinal cord injured Patients: A convenience sample of 10 patients (*N* = 10, 2 female, 8 male; age = 30.1 ± 3.9 years) participated in the study. All included patients sustained high-grade ventral cord impact injuries and had undergone comprehensive clinical and imaging evaluations. At least two clinical assessments for neurologic deficit were performed on each patient by two experienced physicians, the first at presentation, and the second no >1 week prior to performance of the immersion test. For assessment of motor function, each patient was supine decubitus and muscle function was graded from 0 to 5 (Grade 0: absence of muscular activity; Grade 1: palpable or visible contraction; Grade 2: presence of muscle activity (active movement) throughout arch joint movement (full range of motion [ROM]; Grade 3: presence of muscle activity, full ROM, against gravity; Grade 4: presence of muscle activity, full ROM, against gravity and with moderate resistance against an opposing force; and Grade 5: presence of muscle activity, full ROM, against gravity and with strong resistance against an opposing force. For sensory level assessment the two physicians tested pin-prick (pain sensitivity) in every dermatome on both sides of the body. An initial facial stimulus was used as a normal reference and a second stimulus was then made in a cervical, upper limb, thoracic, or lower limb dermatome. Each patient than reported the pain sensitivity of the second stimulus: normal (Grade 2) or altered sensitivity (hypoesthesia or hyperesthesia, Grade 1) or absence of sensitivity (Grade 0). All spinal cord injured patients had both motor and sensory grades of 0 below the determined injury level, compatible with the American Spinal Injury Association Impairment Scale—AIS—A (complete deficit) classification (Maynard et al., 1997). A classification of AIS A is determined by loss of sensory and motor function below the level of injury as well as no preservation of sensory or motor function in sacral segments S4–S5 (Kirshblum et al., 2011). All included patients also had MRI anatomical confirmation of complete cord transection, including 6 with Cervical and 4 with Thoracic cord injuries (Figure 1). In contrast to healthy participants, none of the spinal cord injured patients reported perception of the ice water stimulus. Immersion tests on the patients were performed between 12 and 81 days from injury (mean 47 days).

Cold Water Immersed (±Capsaicin) Healthy participants: Ten healthy adults (*N* = 10, 5 female, 5 male; age = 48.6 ± 5.7 years)

Non-Cold Water Immersed (±Capsaicin) Healthy participants: Ten healthy adults (*N* = 10, 5 female, 5 male; age = 50.3 ± 6.0 years (matching sex and age (±3 years) to cold water immersed group).

Randomization was achieved as follows: for spinal cord-injured patients, a convenience sample of 10 sequential consenting patients was recruited. Limb immersion side was randomized as described below. For volunteer control experiments, the investigators sought expressions of interest from healthy members of their respective hospital or university departments and then assigned blind identifier labels to each volunteer. Ten (5 M, 5 F) were randomly selected for ice-water immersion experiment, and age/sex of each recorded. An additional 10 de-identified volunteers were then selected for warm-water immersion matching sex and age (±3 years). For selection of immersed foot, the first individual in each of the spinal-injured and healthy volunteer ice-water immersion group was assigned foot to be immersed by coin toss. Subsequent participants in each group underwent immersion of the opposite foot. Warm water immersed volunteers had immersed limb side matched to corresponding age and sex matched ice water controls.

A similar method was used for selection of thigh for capsaicin application in the Thermal Sensory Analysis experiment, for which 10 healthy adults (*N* = 10, 5 female, 5 male; age = 49.7 ± 5.8 years were recruited.

Blinding was achieved as follows: for spinal cord-injured patients, the investigators could not be blinded to allocation, but each subject's data, following collection, was de-identified, and analysis was blind. Volunteer participants were provided blind identifier labels for group allocation as described above, but data collection could not be blinded. However, each subject's data, following collection, was de-identified, and analysis was blind.

All participants provided written informed consent prior to participation. The study protocol was approved and overseen by the Institutional Human Ethics Committees of Austin Health, Curtin University and Murdoch University.

### Experimental Procedure

#### Water Immersion Experiments:

All spinal cord injured patients and cold water immersed healthy participants underwent the following procedure: to monitor changes in facial blood flow, laser probes (Moor Instruments, Devon, UK) were attached with adhesive washers to the left and right frontotemporal region in the distribution of branches of the superficial temporal artery. The probes were covered with a headband to reduce interference from random illumination of the recording site and each participant's eyes were covered to prevent laser exposure and also to ensure blinding of spinal cord injured patients to type of immersion. The headband was stretched slightly to hold the probes in place but was not tight enough to interfere with skin blood flow. Signals were transmitted to a laptop PC. After a physiological baseline was established, participants place their left or right foot to ~2.5 cm above the lateral malleolus into a container of water maintained at 32°C for 4 min, then into another container of water maintained at 2°C for 60 s, then back into the 32°C water. This cycle was repeated two more times. Half of the participants placed their right foot into the water and the remainder place their left foot into the water. Spinal cord injured participants were unaware of which limb was immersed.

The non-cold water immersed healthy participants underwent an almost identical procedure, but, instead of cold water, the immersed foot was placed into and removed from the same container of water maintained at 32°C at identical time intervals to the cold water immersed group, i.e., 4 min then 60 s, repeated three times.

Capsaicin: All healthy participants and 2 of the spinal cord-injured patients also underwent a repeat ice water immersion test after application of capsaicin to the lower thigh of the immersed limb 3 months from the initial procedure. Zostrix topical analgesic cream (0.075%) was applied as a 10 cm wide band circumferentially (20 g per application) around the lower thigh of the side to be immersed, with the experiment (immersion) conducted 5 min following application.

All healthy participants experienced burning within 1 or 2 min of application, which then reduced over the next 5 min. The burning then gradually redeveloped over the next 6 h and was maximal about 8 h after application with the lower thigh skin taking on a sunburnt appearance indicating that the capsaicin was (a) absorbed very rapidly, (b) caused early stimulation followed by almost immediate desensitization, and (c) then caused delayed central sensitization (O'Neill et al., 2012).

#### Thermal Sensory Analysis:

The 10 healthy participants underwent measurement of Warm Sensation sensory threshold of lower lateral thigh skin using a TSA-II (Medoc Ltd, Ramat Yishai, Israel). For each participant, the thermode plate was taped to a standard location centered 5 cm proximal to the knee crease in the lower lateral thigh. An initial warm stimulus was applied at 33°C, and temperature were then increased by 0.2°C until the first YES response. Stimuli were then decreased by 0.1°C until a NO was given. Subsequently, the direction changed according to the response: increase for NO and decrease for YES. The step was halved at every direction change. The test was terminated when the step reached 0.1°C. Threshold was determined by taking the mean of last YES and last NO.

Capsaicin: All participants then underwent a repeat Thermal Sensory Analysis 5 min after application of capsaicin (20 g X 0.075% Zostrix topical analgesic cream applied as a 10 cm wide band circumferentially) around the same lower thigh within 7 days of the initial procedure.

#### Skin Biopsies:

At a single sitting, four skin biopsies were obtained without regional anesthesia (to minimize neural damage) from one of the healthy participants who had undergone the ice-water immersion experiment. The volunteer was positioned supine and a circumferential line was drawn 7 cm proximal to the popliteal crease of the opposite thigh to that immersed. Using a 3 mm punch biopsy needle (Kai Medical®, Japan) biopsies were taken along this line at 9 o'clock (lateral), 12 o'clock (dorsal), and 3 o'clock (medial) locations. The participant was then positioned prone and a biopsy was taken at the 6 o'clock (ventral) site. All tissue was fixed in 2% paraformaldehyde with picric acid, embedded in paraffin and serial sagittal 20 μm sections were taken from each block perpendicular to the circumferential line. Sections underwent PgP9.5 immunocytochemistry using accepted techniques. Mounted sections were examined with confocal scanning laser microscopy (Nikon upright *eclipse* 90i).

### Data Analysis

Raw data from the immersion experiments were acquired and analyzed using proprietary software developed by the acquisition instrument's manufacturer (MoorVMS-PCv4.0). At each time point, the instrument computed mean facial flux (proportional to average speed of blood cells x concentration of blood cells) within the sampling area on both sides of each participant's face. Raw data from the thermal sensory analysis experiments were acquired and analyzed using proprietary software developed by the acquisition instrument's manufacturer (Medoc Main Station v6.3).

### Statistics

**Figure 2:**
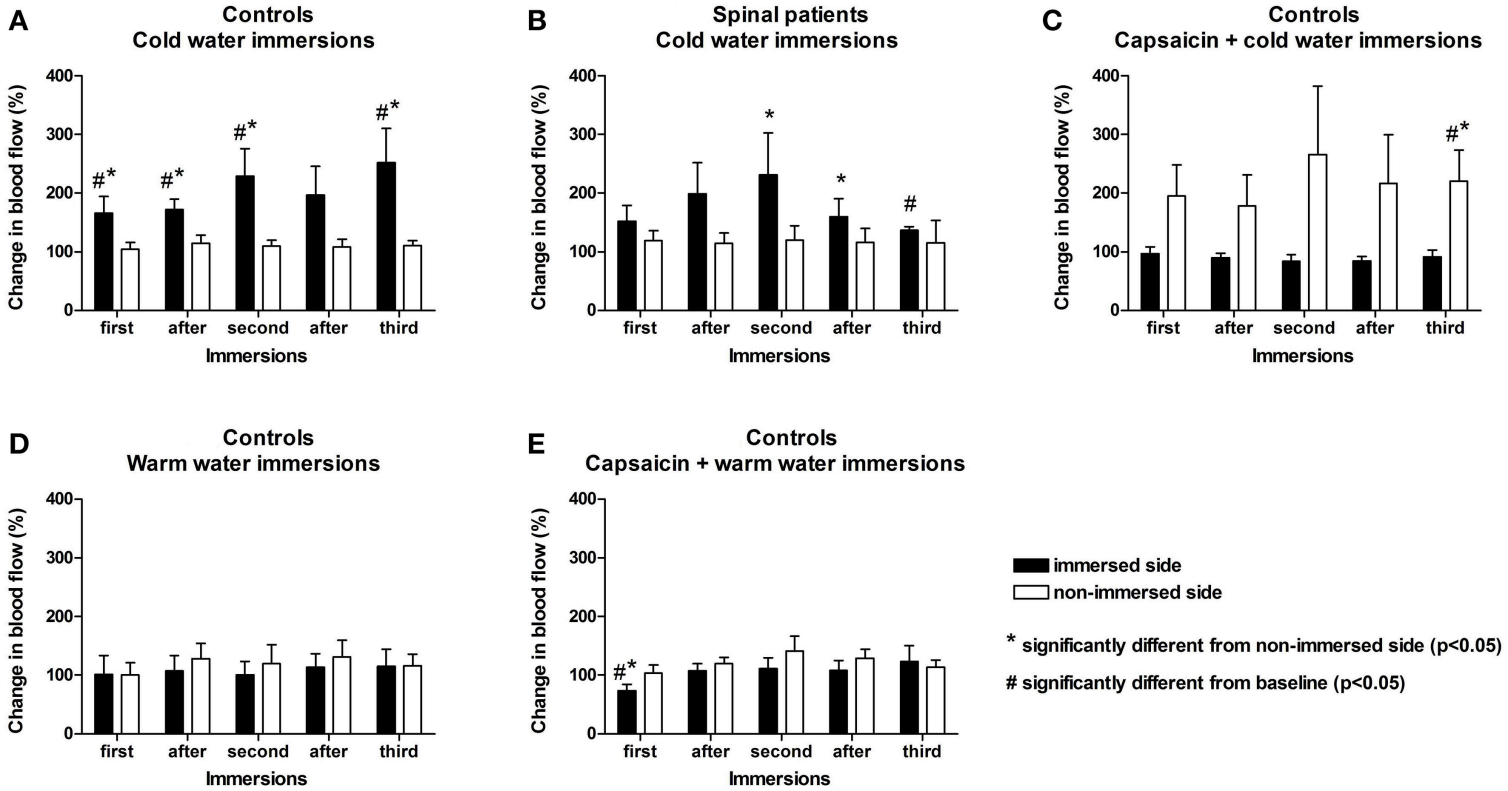
Cold water foot immersion increases facial blood flow. Baseline flux values were obtained after 4 min of warm water immersion. **(A,B)** No difference found between control subjects and spinal cord-injured patients over the 15 min duration of the experiment. Ipsilateral flux intensities increase significantly after initial nociceptive ice immersion in both groups **(C)** Following application of capsaicin cream to lower thigh of the immersed limb prior to cold pressor challenge in healthy controls, ipsilateral facial blood flow remained stable but contralateral facial blood flow increased. **(D,E)** Application of capsaicin to lower thigh of immersed limb did not result in an increase in facial blood flow in healthy controls undergoing warm water immersion only without nociceptive challenge. The small reduction in ipsilateral facial blood flow only identified immediately after capsaicin application may reflect a direct vasoconstrictive effect (Keeble and Brain, 2006; Corbelli et al., 2012). #Change from baseline statistically significant (*p* < 0.05). *Immersed vs. non-immersed side statistically significant (*p* < 0.05).

Statistical analyses were performed with SPSS version 24 and XLSTAT 2017.6. For each condition (immersion experiments), means and standard errors of blood flow were calculated across participants, with standard error indicating inter-subject variability. We conducted repeated measures one and two-factor ANOVA to test for an effect of each immersion across conditions (significance level α = 0.05; SPSS version 24 and XLStat 2017.6). We used paired *t*-tests with Bonferroni correction for multiple comparisons. For the primary outcome measures of the immersion experiments, increase in blood flow, ice water immersion, and capsaicin had significant effects. For the Thermal Sensory Analysis experiments, capsaicin had a significant effect. The immersion results are displayed graphically in Figure 2.

## Results

To determine whether nociceptive information can bypass an interrupted spinal cord, we conducted our previously verified painfully-cold water foot immersion test (Drummond and Chung, 2012) in 10 patients with clinical and imaging confirmation of recent traumatic complete spinal cord injury and compared the results with those of an identical test performed on 10 healthy volunteers. One foot of each participant was immersed in painfully-cold water for a total of 3 min within a 15-min session of data acquisition, with facial blood flow measured by laser Doppler flowmetry (Rajan et al., 2009). To determine whether nociceptive transmission occurs longitudinally in the skin, the 10 healthy participants also underwent the painfully-cold water foot immersion test after application of topical capsaicin to the immersed foot's lower thigh to induce C-fiber conduction blockade (O'Neill et al., 2012). We also measured facial blood flow in another 10 age and gender matched healthy participants both before and after application of topical capsaicin to the lower thigh ipsilateral to the immersed foot to induce C-fiber conduction blockade, but without cold water foot immersion.

### Ice Water Foot Immersion Increases Ipsilateral Facial Blood Flow in Spinal Cord Injured Patients

At the completion of 15 min of data acquisition, ipsilateral facial blood flow had increased by a mean of 76% in spinal cord-injured patients, with no significant difference from the 103% increase in healthy volunteers (Figures 2A,B and Tables 1, 2).

**Table 1:** Cold immersed spinal injured patients' facial flux.

				**4^**′**^**	**5^**′**^**	**9^**′**^**	**10^**′**^**	**14^**′**^**	**15^**′**^**
**Subject**	**Capsaicin**	**Immersed**	**Foot**	**4^**′**^ W**	**1^**′**^ C**	**4^**′**^ W**	**1^**′**^ C**	**4^**′**^ W**	**1^**′**^ C**
1	N	Right	R	39.9	77.3	68.8	62.6	61.9	49.9
			L	55.3	116.1	51.4	39.4	54	41.6
		Imm/Non-imm		0.72	0.67	1.34	1.59	1.15	1.2
2	N	Left	L	105.3	155.1	144.2	120.1	105.8	148.4
			R	20.5	21.4	18.1	24.4	22.2	25.7
		Imm/Non-imm		5.14	7.25	7.97	4.92	4.77	5.77
3	N	Left	L	4	4	4.6	5.8	4	4.6
			R	6.8	4.2	5	5.7	5.9	3.7
		Imm/Non-imm		0.59	0.95	0.92	1.02	0.68	1.24
4	N	Left	L	7.4	10.7	13.5	17.3	14.6	10.1
			R	3.2	3.5	5.3	3.7	3.2	2.3
		Imm/Non-imm		2.31	3.06	2.55	4.68	4.56	4.39
5	N	Right	R	16.7	26.7	88.8	113.2	28.6	23.9
			L	18.5	24	29.3	52.7	28.7	23.8
		Imm/Non-imm		0.9	1.11	3.03	2.15	0.99	1
6	N	Left	L	27.8	21	18	25.1	36	35.9
			R	129.2	66.2	111.8	88	21.4	14.8
		Imm/Non-imm		0.22	0.32	0.16	0.29	1.68	2.43
7	N	Right	R	24.4	90.7	119.8	150.9	103.6	42.1
		Left	L	8.6	17.3	20.2	19.7	25.8	38.4
		Imm/Non-imm		2.84	5.24	5.93	7.66	4.01	1.1
8	N	Right	R	8.3	8.6	8	6.9	9.4	12
			L	5.8	3.7	2.7	2.2	6.3	2.3
		Imm/Non-imm		1.43	2.32	2.96	3.14	1.49	5.22
9	N	Right	R	9.6	11.6	6.6	5.7	8.1	14.7
			L	14.6	19	11.6	11	12.6	15.7
		Imm/Non-imm		0.66	0.61	0.57	0.52	0.64	0.94
10	N	Left	L	57.4	60.4	73.3	77.1	68.2	61.3
			R	83.1	107.2	98.8	92.2	86.2	74.3
		Imm/Non-imm		0.69	0.56	0.74	0.84	0.79	0.83

*Facial flux values at serial time points following each immersion (4 min Warm followed by 1 min Cold, repeated three times) in the 10 spinal cord injured patients who underwent ice cold water immersion. Imm/No-Imm = ratio of immersed side flux to non-immersed side. Ice water immersion resulted in significantly increased ipsilateral facial blood flow between immersed and non-immersed sides (Imm vs. Non-Imm) at 10 and 14 min, and between cold immersed side and baseline (end of initial 4 min of warm) at 15 min*.

**Table 2A:** Cold immersed healthy participants: no-capsaicin.

				**4^**′**^**	**5^**′**^**	**9^**′**^**	**10^**′**^**	**14^**′**^**	**15^**′**^**
**Subject**	**Capsaicin**	**Immersed**	**Foot**	**4^**′**^ W**	**1^**′**^ C**	**4^**′**^ W**	**1^**′**^ C**	**4^**′**^ W**	**1^**′**^ C**
1	N	Right	R	14.5	16.6	23.8	24.9	18.7	18.9
			L	9.9	10.5	13.2	12.2	11.1	12.4
		Imm/Non-imm		1.46	1.58	1.8	2	1.68	1.52
2	N	Left	L	8.4	7.8	22.8	48	53	62.3
			R	4.6	5.9	4.7	5.8	4.7	4.8
		Imm/Non-imm		1.83	1.32	4.85	8.28	11.3	13
3	N	Left	L	8.5	14.3	11.8	28.8	16.8	23.1
			R	5.6	6.2	6.6	6.4	6.8	6.7
		Imm/Non-imm		1.52	2.31	1.79	4.5	2.47	3.45
4	N	Left	L	8.2	7.8	8.3	8.2	9.1	14.5
			R	43.5	34.1	27.5	21.7	32.5	53.9
		Imm/Non-imm		0.19	0.23	0.3	0.38	0.28	0.27
5	N	Right	R	17.3	51.8	29.4	25.5	25.2	42.6
			L	13.4	9.9	7.7	8.4	11.6	8.5
		Imm/Non-imm		1.29	5.23	3.82	3.03	2.17	5.01
6	N	Left	L	8.3	20.8	17.3	17.2	12.8	10.3
			R	5.7	7	9.4	7.8	9.1	6.7
		Imm/Non-imm		1.45	2.97	1.84	2.21	1.41	1.54
7	N	Right	R	18.4	17.2	27.8	45.5	26.7	36.8
			L	27.5	12.1	23.9	33.1	20.4	23.4
		Imm/Non-imm		0.67	1.42	1.16	1.37	1.31	1.57
8	N	Right	R	12.4	12	12.1	10.9	12.3	11.9
			L	12.7	10.1	11.8	9.9	11.2	11.7
		Imm/Non-imm		0.94	1.19	1.02	1.1	1.1	1.2
9	N	Right	R	49.9	63.8	90.5	59.4	84.1	144.8
		Left	L	24	34.1	48.2	30.7	46.8	38.1
		Imm/Non-imm		2.08	1.87	1.88	0.232	1.8	3.8
10	N	Left	L	7.4	23.6	17.6	22.2	13.7	18.1
			R	24.4	39.7	29.5	37.8	16.6	27.9
		Imm/Non-imm		0.3	0.59	0.6	0.59	0.83	0.65

*Facial flux values at serial time points following each immersion (4 min Warm followed by 1 min Cold, repeated three times) in 10 healthy participants who underwent ice cold water immersion, both before **(A)** and after **(B)** capsaicin application to lower thigh of immersed foot. Imm/No-imm = ratio of immersed side flux to non-immersed side. Ice water immersion resulted in significantly increased ipsilateral facial blood flow between immersed and non-immersed sides (Imm vs. Non-imm) and between cold immersed side and baseline (end of initial 4 min of warm) at 5, 9, 10, and 15 min*.

**Table 2B:** Cold immersed healthy participants: post-capsaicin.

				**4^**′**^**	**5^**′**^**	**9^**′**^**	**10^**′**^**	**14^**′**^**	**15^**′**^**
**Subject**	**Capsaicin**	**Immersed**	**Foot**	**4^**′**^ W**	**1^**′**^ C**	**4^**′**^ W**	**1^**′**^ C**	**4^**′**^ W**	**1^**′**^ C**
1	Y	Right	R	47.2	66.6	42	31	32.5	45
			L	21.6	132.6	136.2	282.4	206.1	134.2
		Imm/Non-imm		2.19	0.5	0.31	0.11	0.16	0.33
2	Y	Left	L	17.8	7.8	12.1	7.3	16.9	11.1
			R	10.4	8.7	10.2	12.6	11.6	10.2
		Imm/Non-imm		1.71	0.9	1.18	0.58	1.46	1.09
3	Y	Left	L	6.5	6.5	7.1	7.3	6.9	7
			R	6.7	6.8	7	10.2	10.3	8.4
		Imm/Non-imm		0.97	0.96	1.01	0.72	0.67	0.83
4	Y	Left	L	24.7	40.4	28.4	26.1	28	28.1
			R	24.4	45	32.6	31.5	31.7	30.7
		Imm/Non-imm		1.01	0.9	0.87	0.84	0.88	0.92
5	Y	Right	R	74.2	71	62.8	63.1	68.3	61.6
			L	7.3	9.7	8.7	9.3	10	8.2
		Imm/Non-imm		10.2	7.32	7.22	6.78	6.83	7.51
6	Y	Left	L	80.1	43.4	53.4	47.6	60.5	75.6
			R	38.6	139.4	98.5	93.4	95.3	149.3
		Imm/Non-imm		2.08	0.31	0.54	0.51	0.63	0.51
7	Y	Right	R	7	6.5	9.2	10.4	8.4	12.3
			L	8.4	10.3	8.3	10	8.3	20.5
		Imm/Non-imm		0.83	0.63	1.11	1.04	1.01	0.6
8	Y	Right	R	12.2	13.9	11.2	5.2	5.7	5.7
			L	7.4	7.3	6.2	5	7.9	17.8
		Imm/Non-imm		1.65	1.9	1.81	1.04	0.72	0.32
9	Y	Right	R	38.9	26.7	20.6	20	19.3	19.7
		Left	L	55.6	86.8	88.7	123.3	63.3	57.5
		Imm/Non-imm		0.7	0.31	0.23	0.16	0.3	0.34
10	Y	Left	L	86.8	81.6	78.8	106	64.1	70.6
			R	94.2	88.7	92.6	158	110.4	139.7
		Imm/Non-imm		0.92	0.92	0.85	0.67	0.58	0.51

*Facial flux values at serial time points following each immersion (4 min Warm followed by 1 min Cold, repeated three times) in 10 healthy participants who underwent ice cold water immersion, both before **(A)** and after **(B)** capsaicin application to lower thigh of immersed foot. Imm/No-Imm = ratio of immersed side flux to non-immersed side. Ice water immersion after capsaicin resulted in significantly increased contralateral facial blood flow between immersed and non-immersed sides (Imm vs. Non-imm) and between cold immersed side and baseline (end of initial 4 min of warm) at 15 min*.

### Capsaicin Causes Rapid Cutaneous C-Fiber Conduction Blockade

As ice-water foot immersion increased ipsilateral facial blood flow in both healthy participants and spinal cord-injured patients, we suspected the existence of a cutaneous C-fiber pathway running longitudinally along the ipsilateral leg, thigh, abdominal, thoracic, and neck skin, capable of bypassing a transected cord. To confirm such a pathway, we sought to create a method to selectively block C-fibers at a level sufficiently distant from the foot (where fibers conveying normally perceived sensory input transmit through tibial and peroneal nerves). Given that we needed to circumferentially block all cutaneous C-fiber transmission, we chose the lower thigh for application of a blocking agent as proximal sites (such as chest or abdomen) would require much higher doses, while the neck was deemed impractical and of greater risk.

Lidocaine and related sodium channel blocking agents are widely used drugs for cutaneous neural blockade (Leppert et al., 2018), and we initially contemplated using the long-acting local anesthetic cream, EMLA, but were deterred for 2 reasons:

(i) EMLA is not without side effects, and, given the large volume of skin that we needed to anesthetize, there was potential for systemic absorption. There are reports of cardiac toxicity (Tran and Koo, 2014) and other serious adverse reactions (Hahn et al., 2004) from administration of large quantities of EMLA.

(ii) EMLA's effect is non-selective: it also blocks sympathetic efferents, resulting in both local vasoconstriction and vasodilation (Bjerring et al., 1989), which could have, potentially, confounded our attempt to assess increases in blood flow. Similarly, had we employed EMLA, which non-selectively blocks all sensory fibers, we would not have been able to distinguish between C-fibers (our proposed neural pathway) and other afferent (i.e., A-fiber) categories (Vallbo et al., 1979).

**Figure 3:**
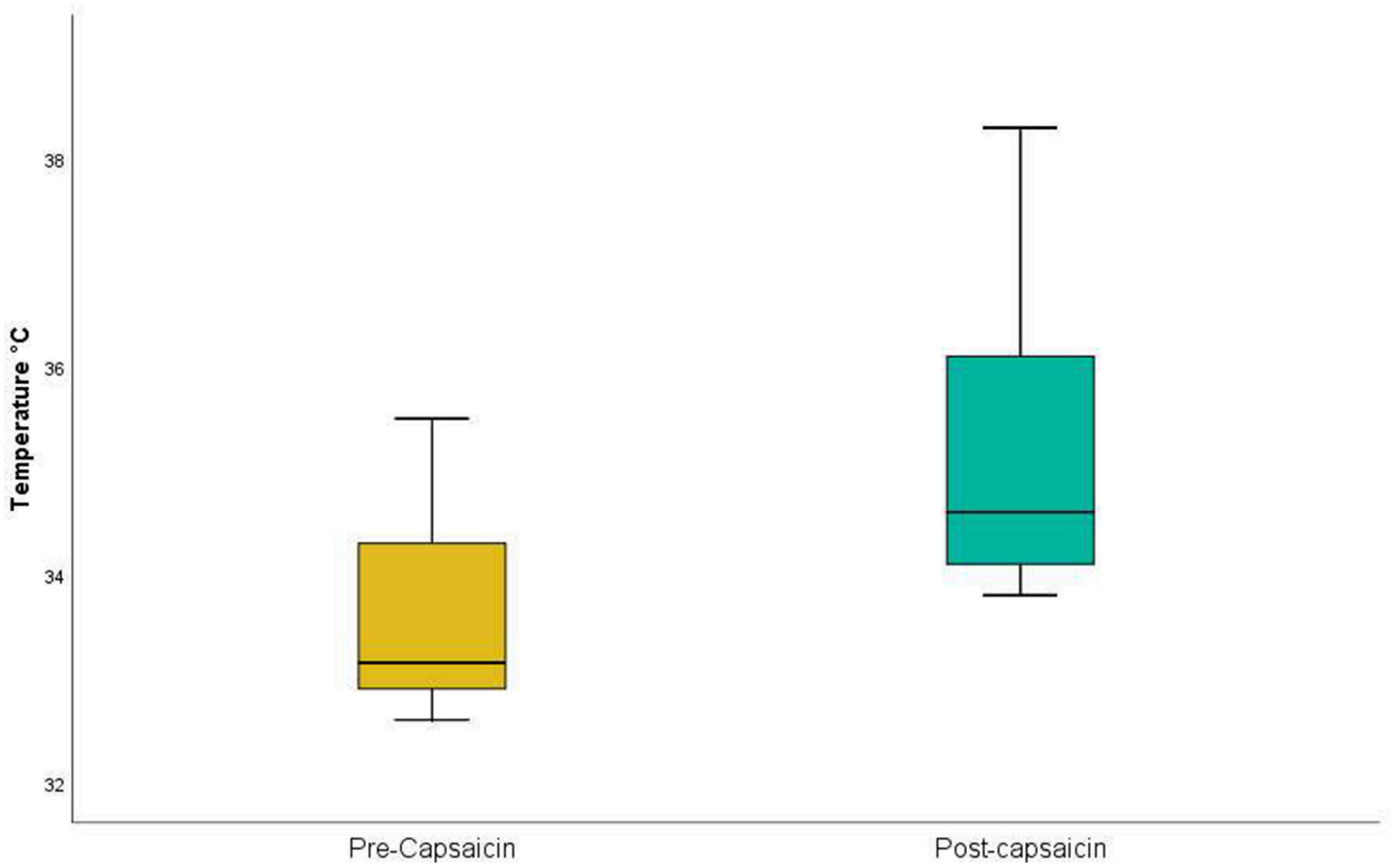
Thermal Sensory Analysis in healthy participants: pre- and post-capsaicin. Application of capsaicin to lower thigh significantly (*p* = 0.00001) increased heat detection threshold in the lower thigh.

Capsaicin, in contrast, acts selectively on C-fiber afferents (O'Neill et al., 2012) and is deemed to be safe. Acute desensitization is induced within 20 s of neural exposure to capsaicin (Novakova-Tousova et al., 2007) and significant capsaicin concentrations are present within the human epidermis within 1 min of topical application (Pershing et al., 2004). While capsaicin causes delayed effects, including peripheral and central sensitization (O'Neill et al., 2012), we reasoned that it would be a suitable agent for short-term C-fiber desensitization, and sought to confirm this in a trial on 10 volunteer health participants. Employing a Thermal Sensory Analyzer (Yosipovitch and Maibach, 1998) for detection of a warm stimulus (that is selective for C-fibers: Hensel et al., 1960; Mackenzie et al., 1975), we confirmed that the application of capsaicin to the lower thigh significantly increased heat detection threshold (Table 3 and Figure 3). While it is possible that burning sensations from the capsaicin might have masked more subtle sensations of warmth, all participants indicated that warmth perceived from the capsaicin had subsided by the time thermal sensory analysis was commenced. Hence, the application of capsaicin to lower thigh skin likely causes rapid cutaneous C-fiber conduction blockade.

**Table 3:** Thermal Sensory Analysis in healthy participants: pre- and post-capsaicin.

**Subject**	**Gender**	**Age**	**Limb**	**Pre-capsaicin**	**Post-capsaicin**
1	M	57	L	34.4	36.4
2	F	18	R	32.6	33.8
3	F	48	L	33	34.1
4	M	21	R	32.7	34.4
5	F	47	L	34.1	35.5
6	F	71	R	33.3	34.2
7	M	73	L	34.3	36.1
8	F	45	R	35.5	38.3
9	M	60	L	32.9	34
10	M	57	R	32.9	34.8

*Application of capsaicin to lower thigh significantly (p = 0.00001) increased heat detection threshold in the lower thigh*.

### Capsaicin C-Fiber Blockade Increases Contralateral Facial Blood Flow After Ice Water Foot Immersion

Immersion of the foot after application of capsaicin in healthy volunteers caused a significant progressive increase in contralateral facial blood flow, which had increased by a mean of 111% at completion of 15 min of data acquisition (Figure 2C and Table 2). Capsaicin did not result in any difference in facial blood flow in the absence of painfully-cold water foot immersion (Figures 2D,E and Table 4). Two of our original cohort of spinal cord-injured patients gave consent to repeating the ice water immersion experiment following topical capsaicin application to the lower thigh of the immersed foot. Facial blood flow increased on the ipsilateral side in one patient and did not change substantially on either side in the other, in contrast to the contralateral increase induced by capsaicin in healthy participants (Table 5).

**Table 4A:** Warm immersed healthy participants: no-capsaicin.

				**4^**′**^**	**5^**′**^**	**9^**′**^**	**10^**′**^**	**14^**′**^**	**15^**′**^**
**Subject**	**Capsaicin**	**Immersed**	**Foot**	**4^**′**^ W**	**1^**′**^ C**	**4^**′**^ W**	**1^**′**^ C**	**4^**′**^ W**	**1^**′**^ C**
1	N	Left	L	23.4	18.8	21.4	19.3	24.6	22.7
			R	22.7	6.5	7.4	5.8	7	8.5
		Imm/Non-imm		1.03	2.89	2.89	3.33	3.5	2.7
2	N	Right	R	17.6	9.3	15	16.1	13.7	12.8
			L	15.9	19.8	24.8	25.1	25.1	24.1
		Imm/Non-imm		1.11	0.47	0.6	0.64	0.55	0.53
3	N	Left	L	65.9	55.5	60.1	54	57.4	53.8
			R	48.7	37.7	37.5	39.6	39.4	40.6
		Imm/Non-imm		1.35	1.47	1.6	1.36	1.46	1.32
4	N	Right	R	62.8	59.6	40	35.5	34.1	33.9
			L	33.6	31.7	27.2	30.8	41.3	46.4
		Imm/Non-imm		1.87	1.88	1.47	1.15	0.83	0.73
5	N	Right	R	30.9	18.5	13.3	13.2	14.9	13.8
			L	32.7	22.5	19.3	16.6	21.3	19.5
		Imm/Non-imm		0.94	0.82	0.69	0.8	0.7	0.71
6	N	Left	L	63.7	35.2	24.8	24.6	79.9	88.9
			R	117.7	29.3	41.2	52.2	80.7	67
		Imm/Non-imm		0.54	1.2	0.6	0.47	0.99	1.33
7	N	Right	R	28.6	111.3	45.3	17.5	19.8	26.3
			L	79.7	204.1	143.4	61.6	57.1	93
		Imm/Non-imm		0.36	0.55	0.32	0.28	0.35	0.28
8	N	Left	L	14.2	4.7	7.8	22.9	29	28.1
			R	15.5	22.1	41.5	25.5	42.7	39.7
		Imm/Non-imm		0.92	0.21	0.19	0.9	0.68	0.71
9	N	Right	R	24.6	20.6	78.1	67.7	68	83.3
			L	50.6	63.2	82.3	60.3	71.2	66.2
		Imm/Non-imm		0.49	0.33	0.95	1.12	0.96	1.26
10	N	Left	L	76.7	61.4	98	87.8	63.8	21.9
			R	36.7	21.9	83	140.3	109.2	44.9
		Imm/Non-imm		2.1	2.8	1.18	0.63	0.58	0.49

*Facial flux values at serial time points following each immersion (4 min Warm followed by 1 min Cold, repeated three times) in 10 healthy participants who underwent warm water immersion only, both before **(A)** and after **(B)** capsaicin application to lower thigh of immersed foot. Imm/No-Imm = ratio of immersed side flux to non-immersed side. There were no significant differences between sides prior to capsaicin application. Capsaicin resulted in significantly decreased ipsilateral facial blood flow between immersed and non-immersed sides (Imm vs. Non-imm) at baseline (end of initial 4 min of warm), but had no impact on facial blood flow at later time points*.

**Table 4B:** Warm immersed healthy participants: post-capsaicin.

				**4^**′**^**	**5^**′**^**	**9^**′**^**	**10^**′**^**	**14^**′**^**	**15^**′**^**
**Subject**	**Capsaicin**	**Immersed**	**Foot**	**4**″** W**	**1^**′**^ C**	**4^**′**^ W**	**1^**′**^ C**	**4^**′**^ W**	**1^**′**^ C**
1	Y	Left	L	34.3	13.3	35.1	53.2	49.7	62.9
			R	12.6	21	22	38	18	14.5
		Imm/Non-imm		2.72	4.89	1.6	1.4	2.72	4.34
2	Y	Right	R	7.3	7.6	14.2	19.3	15.5	18.3
			L	7.3	7.2	10.6	18.5	14.1	14.6
		Imm/Non-imm		1	1.06	1.34	1.04	1.11	1.25
3	Y	Left	L	145.9	118.9	109.2	85.8	75.6	59.8
			R	50.5	56.4	58	52.3	61	55.5
		Imm/Non-imm		2.96	2.11	1.88	1.64	1.24	1.08
4	Y	Right	R	34.8	15	32.4	31.6	24.8	25.2
			L	48.8	25.7	54.8	56.4	42.5	47.6
		Imm/Non-imm		0.71	0.58	0.59	0.56	0.58	0.53
5	Y	Right	R	13.4	11.1	11.4	10.2	16.8	37
			L	17.3	17.1	21.2	22.3	33.9	20.1
		Imm/Non-imm		0.77	0.65	0.54	0.46	0.5	1.85
6	Y	Left	L	60.9	78	86.8	65.2	47	64.1
			R	82.3	156.7	128.8	97.4	86.2	69.1
		Imm/Non-imm		0.74	0.5	0.67	0.67	0.55	0.93
7	Y	Right	R	14.2	7.3	7.1	7	6.7	6.7
			L	16.1	12.5	11	10.8	12.1	13.2
		Imm/Non-imm		0.88	0.58	0.65	0.65	0.55	0.51
8	Y	Left	L	33.7	28.5	26.9	36.8	24.9	14.1
			R	53	52.3	54.5	88	96.1	85.8
		Imm/Non-imm		0.64	0.54	0.49	0.42	0.26	0.16
9	Y	Right	R	81.5	76.4	89.4	75.6	78.2	71.8
			L	59.5	54.8	71.9	69.4	75.8	60.5
		Imm/Non-imm		1.37	1.39	1.24	1.09	1.03	1.19
10	Y	Left	L	63.4	17.5	85.8	63.8	111.6	78.3
			R	120.2	52.8	94.7	43.1	66.1	78.5
		Imm/Non-imm		0.53	0.33	0.91	1.48	1.69	1

*Facial flux values at serial time points following each immersion (4 min Warm followed by 1 min Cold, repeated three times) in 10 healthy participants who underwent warm water immersion only, both before **(A)** and after **(B)** capsaicin application to lower thigh of immersed foot. Imm/No-Imm = ratio of immersed side flux to non-immersed side. There were no significant differences between sides prior to capsaicin application. Capsaicin resulted in significantly decreased ipsilateral facial blood flow between immersed and non-immersed sides (Imm vs. Non-Imm) at baseline (end of initial 4 min of warm), but had no impact on facial blood flow at later time points*.

**Table 5:** Cold immersed spinal cord injured-patients: post-capsaicin.

				**4^**′**^**	**5^**′**^**	**9^**′**^**	**10^**′**^**	**14^**′**^**	**15^**′**^**
**Subject**	**Capsaicin**	**Immersed**	**Foot**	**4^**′**^ W**	**1^**′**^ C**	**4^**′**^ W**	**1^**′**^ C**	**4^**′**^ W**	**1^**′**^ C**
1	Y	Right	R	14.4	17.5	57.6	72.7	41.9	63.1
			L	19.6	25.2	70.1	79.8	47.6	48.8
		Imm/Non-imm		0.73	0.69	0.82	0.91	0.88	1.29
9	Y	Right	R	35.8	40	27.8	37.2	40.9	49.9
			L	44.9	62.7	43.7	40.7	40.9	58.9
		Imm/Non-imm		0.8	0.64	0.64	0.91	1	0.85

*Facial flux values at serial time points following each immersion (4 min Warm followed by 1 min Cold, repeated three times) in 2 of the spinal cord-injured patients who underwent ice cold water immersion, following capsaicin application to lower thigh of immersed foot. Imm/No-Imm = ratio of immersed side flux to non-immersed side*.

**Figure 4:**
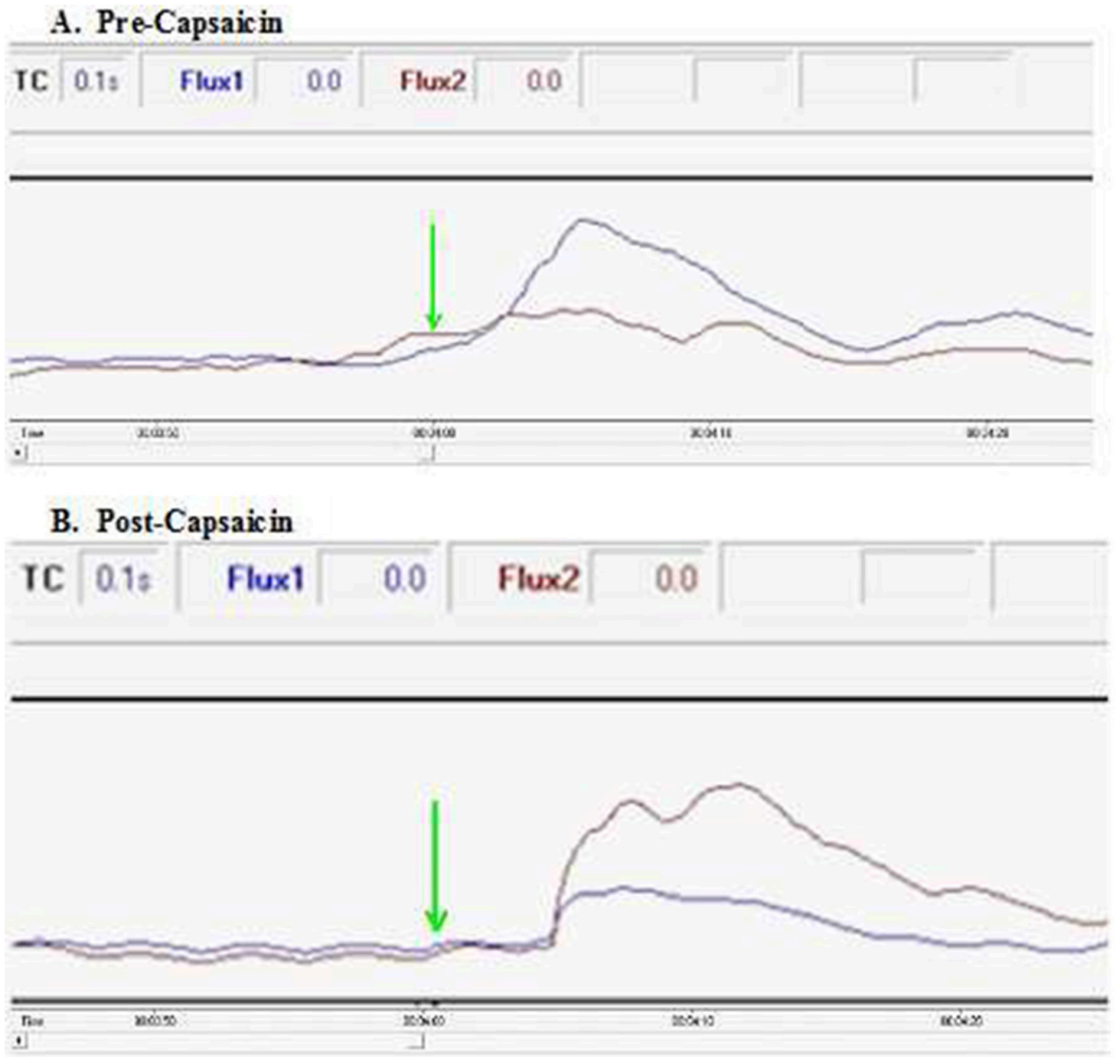
Excerpts of flux spectra pre- and post-capsaicin in a healthy participant. Conduction velocity was estimated using a time point of first doubling of ipsilateral blood flow following the start of the initial cold water immersion at 4 min (green arrow). **(A)** Ipsilateral facial blood flow (blue) doubles within the first 3 s following ice water immersion. In contrast, application of capsaicin around the lower thigh of the immersed limb **(B)** results in an increase in contralateral facial blood flow (red). In both spectra, a smaller early rise occurs in blood flow to the opposite side of the face, presumably due to axon reflex propagation (also see Supplementary Videos 1, 2).

To further characterize the neurophysiology of this pathway connecting the foot to ipsilateral facial skin, we estimated conduction velocity in the 10 healthy participants undergoing painfully-cold water foot immersion using a time point of first doubling of ipsilateral blood flow following the start of the initial cold water immersion at 4 min (Figure 4). Estimated mean velocity of 0.46 ± 0.15 m/s is at the lower range, but still consistent, with those previously reported for cutaneous nociceptive C-fiber afferents (Serra et al., 1999). Notably, our velocity estimate is well below the described velocity for spinothalamic tract C-fiber pain transmission of around 2.2 m/s (Qiu et al., 2001), in keeping with an extra-spinal transmission path.

### C-Fiber Branch Site Is Located in Subepidermal Thigh Skin

**Figure 5:**
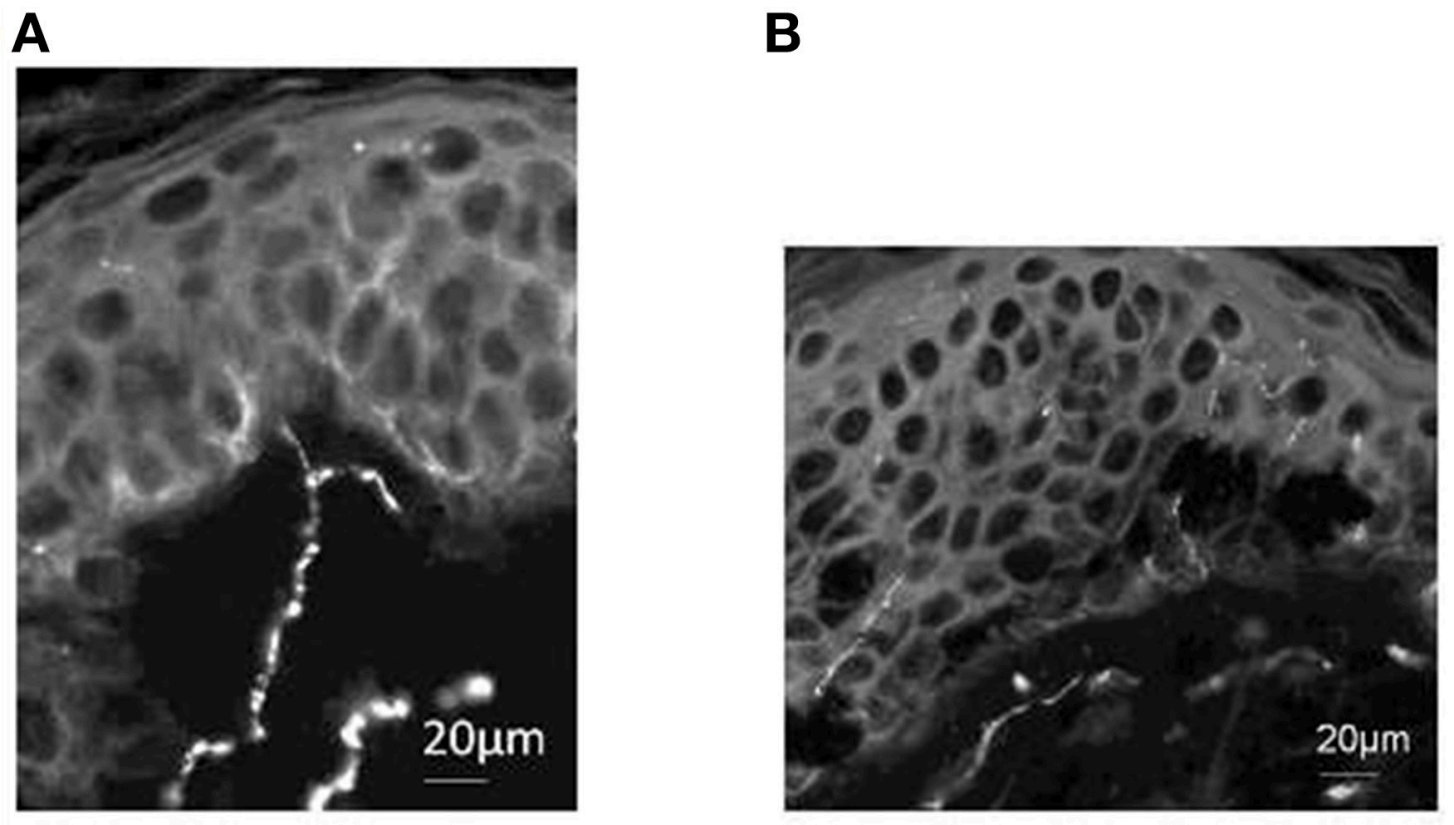
PgP9.5 immunostaining of skin from healthy volunteer demonstrates subepidermal axonal branch site. **(A)** Section from lateral thigh skin includes a single branching PgP9.5 positive axon just beneath the epidermis. **(B)** Section from medial thigh skin demonstrates intra-epidermal and sub-epidermal axons but no additional branch sites were identified at this or other 2 sites sampled. Scale bars: **(A)**; 20 μm **(B)**; 20 μm.

One of our volunteer participants who underwent painfully cold-water foot immersion consented to undergo four separate skin biopsies of the lower thigh opposite to that immersed. Using PgP9.5 immunostaining as a neuronal marker (Dalsgaard et al., 1989), we sought evidence of bifurcating axons in sub-epidermal skin. As illustrated in Figure 5, a single branching PgP9.5 positive axon just beneath the epidermis was present on one lateral thigh skin section. We did not find any other axonal branch sites on any of the other sections.

## Discussion

**Figure 6:**
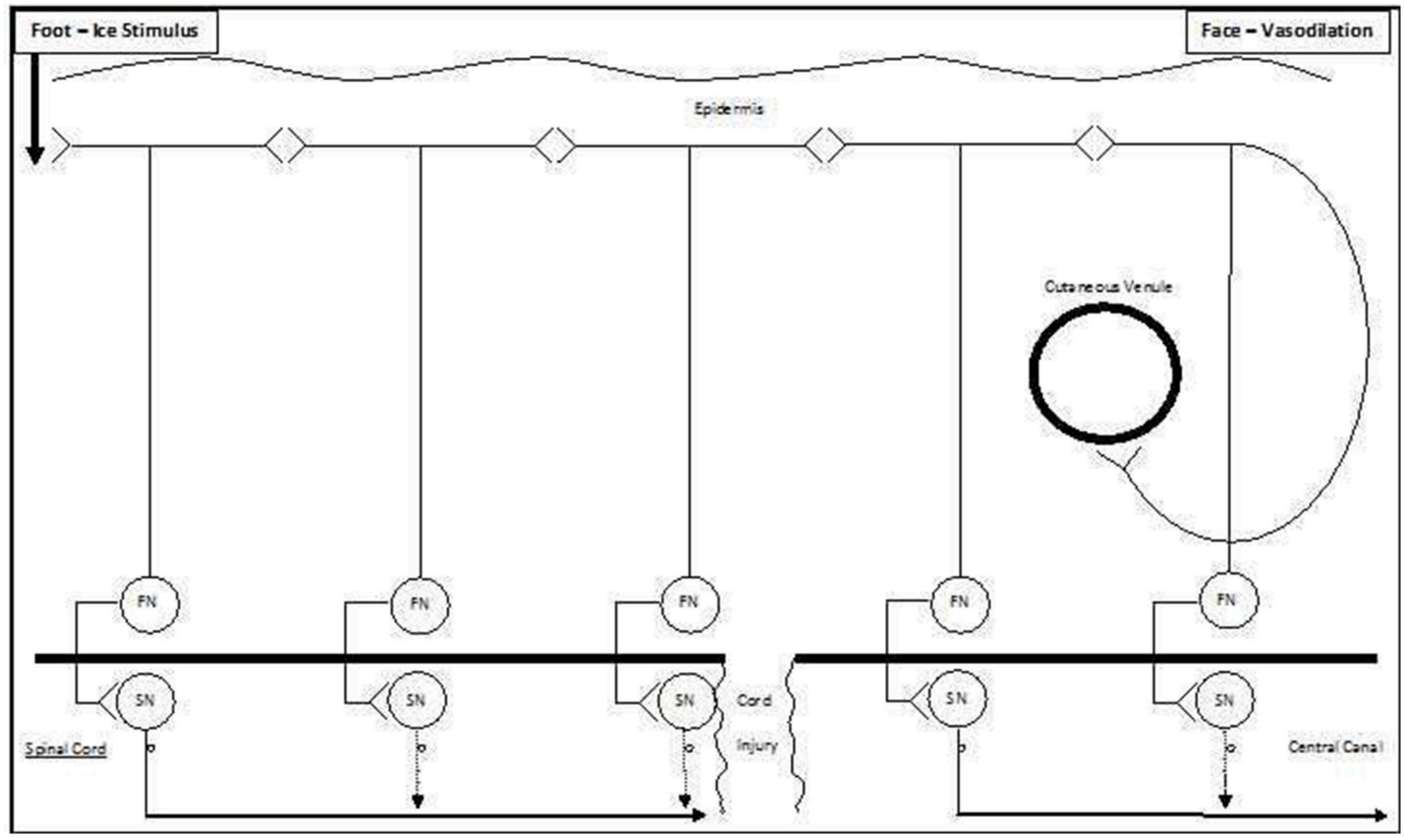
Schematic model of ipsilateral vasodilation occurring following ice water foot immersion. We suggest that the preferred pathway for ipsilateral extracranial vasodilation following foot immersion is via a series of antidromic-orthodromic cutaneous connections (top of Figure). As this pathway partially bypasses the spinal cord, it remains intact following spinal cord injury (bottom of Figure).

Given that the pathway was blocked by ipsilateral lower thigh capsaicin, we suggest that it runs longitudinally in the sub-epidermal skin [akin to that previously described by Zhang et al. (2008)], as a series of paracrine-like C-fiber communications between bifurcating cutaneous sensory nerves, with serial antidromic and orthodromic conduction (Figure 6 and Supplementary Video 1) and is thus amenable to blockade by cutaneous capsaicin (Supplementary Video 2). In Zhang et al. model 2008, neurogenic propagation involves peripheral cross-talk between cutaneous C-fibers and is abolished by capsaicin. The published velocity by Zhang et al. (2008) for this pathway was 1.21 m/s, but it is quite possible that the extension of this model over multiple sites of cross-talk could reduce conduction speed, and this is supported by our mean velocity result of 0.46 m/s. The penultimate paracrine-like connection of this pathway would then be to a sympathetic efferent terminating on a small cutaneous facial venule (Johnson et al., 2005). As noted by Jänig (1989), sympathetic nerves contribute to both vasoconstrictor and vasodilator responses, and, in contrast to non-facial skin, these efferents in facial skin result in vasodilation mediated by beta-adrenergic receptors (Pegram et al., 1976). While there is also simultaneous sensory transmission via deeper nerves and the spinal cord to the contralateral cerebral cortex resulting in perception of the cold pressor stimulus, the cutaneous pathway is shorter, resulting in earlier facial vasodilation (Supplementary Video 1). The cutaneous pathway is unaffected in patients with spinal cord transection, and the cold pressor stimulus still results in ipsilateral facial vasodilation (Supplementary Video 3).

Are there other neurohumeral cutaneous pathways that could explain our results? Bowsher et al. (2009) described a novel form of neurovascular innervation in 2 patients with congenital absence of pain and hyperhidrosis and absent C-fibers. However, these nerves were cholinergic, and as noted by Nolano et al. (2010), capsaicin did not abolish this form of innervation. In contrast, the application of capsaicin to participants' lower thighs in our study did abolish transmission, suggesting that C-fibers (and Substance P) were involved. Could this pathway lie elsewhere, apart from the skin? Sensory afferents are present in the lumbar sympathetic chain, but their functional role is unclear (Jänig and McLachlan, 1986), and it seems unlikely that they would alter excitability further proximally in the cervical sympathetic chain. Our previous work has shown that blocking sympathetic adrenergic activity with guanethidine blocked the ipsilateral vasodilator response in the temple during hand immersion in ice-water, suggesting that vasodilatation was mediated by release of sympathetic vasoconstrictor tone (a reduction in the release of noradrenaline) or that the guanethidine blocked an active vasodilator mechanism (Drummond, 2006). Ice water immersion does induce intense vasoconstriction which is relieved intermittently by release of vasoconstrictor tone (cold-induced vasodilatation or the so-called “hunting” reaction) to inhibit frostbite (Daanen, 2003). Could the “hunting” reaction extend ipsilaterally via the sympathetic chain and the burning sensations induced by capsaicin on the lower thigh cause the response to change sides? Capsaicin application, even in the absence of nociceptive challenge, can induce local blood flow changes, including transient vasoconstriction (Grönroos et al., 1994; Keeble and Brain, 2006), as evidenced by the early small reduction in ipsilateral facial blood flow in our healthy participants who only underwent warm water immersion (Figure 2E). However, the “hunting” reaction is generally delayed following cold exposure and intermittent (Daanen, 2003), in contrast to our demonstration of an immediate effect of ice water immersion and sustained increase in contralateral facial blood flow following capsaicin. If this pathway lay in the sympathetic trunk, presumably undamaged following spinal cord injury, it would not be affected by ipsilateral lower thigh capsaicin application.

Could activation of the parasympathetic nervous system explain our results? Foot cold stimulation might activate local segmental spinal sympathetic reflexes, potentially causing changes in the physiology of visceral organs, then activating the vagus and modulating the autonomic nervous system, leading to facial blood flow changes. Kametani et al. (1979) described vagal-mediated gastric motility enhancement following hindpaw stimulation in rats, presumably due to afferent neural convergence (Qin et al., 2007). In addition, afferent vagal stimulation can modulate sympathetic activity (Schwartz et al., 1973), although there are no known vagal afferents in lower limb skin. Should such a pathway be responsible for our results, this would require lower limb C-fiber afferents to ascend via the sympathetic trunk to synapse with vagal efferent brainstem cell bodies and either directly or via sympathetic modulation, effect facial vasodilation. However, trigeminal-parasympathetic vasodilator reflexes in facial skin are confined to the oral, ocular and possibly nasal regions (Drummond, 1995), outside the area of placement of our detector probes, and thus not detectable in our experiments. In addition, if cold water stimulation of the foot activated vagal afferents, this might be expected to induce bilateral—rather than ipsilateral—changes in facial blood flow. Parasympathetic fibers cross over in the brainstem (Lambert et al., 1984), and in the rat (Suzuki and Hardebo, 1991), cat (Akiyama and Yamazaki, 2001), and presumably human—each sphenopalatine ganglion results in bilateral innervation. While we previously proposed a supraspinal mechanism as a possible explanation for ipsilateral extracranial vasodilatation (Drummond and Chung, 2012), we discount this as all of our spinal-cord injured patients had eyes covered during the experiment, and none could identify which side was immersed when asked.

Could our assumption—that all included spinal cord-injured patients had complete deficits—be erroneous? While all included patients had comprehensive clinical and imaging assessments indicating complete deficits, it remains possible that some sensory pathways could have been preserved, potentially accounting for maintained ipsilateral facial vasodilation. Yet, nociceptive afferents ascend in the opposite anterolateral tracts (Almeida et al., 2004) and would, uniformly, be disrupted in ventral cord impact injuries as occurred in our cohort. Indeed, Noordenobos and Wall (1976) described a patient who had a sub-complete T3 spinal cord transection, with preservation of only one anterolateral quadrant who was able to still perceive some contralateral sensory stimuli below the lesion, but, crucially, could not detect thermal stimuli, which are transmitted by C-fibers (Mackenzie et al., 1975). Finally, at the level of injury, fibers cross the anterior cord transversely (Nathan et al., 2001) and would be expected to be disrupted in lesser-degree injuries than the catastrophic impacts in our patient group. Notably, whether our spinal cord-injured patients had any preserved fibers crossing the level of transection or not, the results of our capsaicin experiments indicate that a longitudinal C-fiber pathway lies outside the spinal cord.

We must also acknowledge limitations when drawing conclusions from our results. In relation to Methods, the arm of our study performed upon spinal cord injured patients included only a small number, as the rigorous inclusion criteria for this study made it difficult to recruit suitable patients able and willing to consent to participation. Yet, similar studies on spinal cord injury by Donati et al. (2016) and Wrigley et al. (2018) have also drawn conclusions based upon small patient numbers, and our results did achieve statistical significance. In addition to repeating this work on larger numbers, there are several other techniques for facial blood flow measurement that could be implemented in future efforts to verify our findings, including LDI (Hoeksema et al., 2014), photoplethysmography (Drummond and Chung, 2012), and thermography (Shearn et al., 1990). Finally, we note that our choice of pharmacologic agent for cutaneous C-fiber blockade, capsaicin, is not completely ideal, given that it induces delayed central sensitization (O'Neill et al., 2012), but lidocaine is also not ideal as we note in our Results, and none of our participants were symptomatic until well after completion of data collection.

Our demonstration that the sensory pathway by which a noxious stimulus applied to the foot can bypass an interrupted spinal cord explains Nathan's original observation (Villanueva et al., 2006) as well as more recent work (Wrigley et al., 2018). While the entity referred to as jogger's migraine (Corbelli et al., 2012) might be explained by this pathway (although not the preceding aura), its end-point—facial vasodilation mediated by beta-adrenergic receptors—supports recent work suggesting a far greater contribution of neurovascular dysfunction to migraine pathophysiology via antidromic vasodilation (Geppetti et al., 2012) and the mechanism by which beta-blockers prevent attacks (Jacobs and Dussor, 2016). This pathway also identifies one potential mechanism for some of the positive effects of acupuncture [often applied to the ipsilateral foot (Silberstein, 2009)] in treating episodic migraine, and may have additional implications for the mechanism by which acupuncture exerts its effect (Linde et al., 2016). Our proposed pathway is also consistent with the neurogenic inflammation-like propagation of acupuncture along meridians recently described in labeling studies by Kim et al. (2017). The clinical entity of referred itch or *mitempfindung* is also an ipsilateral phenomenon, but its mechanism has previously defied explanation (Richter, 1977), yet the pathway we have described readily accounts for its features (Silberstein, 2012). Indeed, it may even be possible to verify our findings by recruiting human volunteers who experience referred itch and exploring whether there is a relationship between lower limb stimulation and facial referral points. Finally, the demonstration of this pathway may have implications for treating patients with spinal cord injuries. Long-term motor training has recently been shown to result in neurological improvement in a small cohort of patients with complete spinal cord injury, but the mechanism by which this occurred could not be explained (Donati et al., 2016). Peripheral cross-talk between C-fiber afferents, the proposed basis for the pathway we have demonstrated, has previously been shown to rely on a series of paracrine-like communications at NK-1 receptors, employing Substance P (SP) (Zhang et al., 2008). Given that SP is responsible for Long Term Potentiation (Sandkühler, 2007), the pathway might be amenable to long-term training by, for example, repeated lower limb noxious stimulation. In the event that our proposed mechanism is confirmed by others, we speculate that it may 1 day be possible to train spinal cord-injured patients to recognize ipsilateral facial vasodilation as an indicator of foot impact on the ground, as part of training to re-establish locomotion.

In conclusion, based upon facial perfusion measurements following painfully-cold water foot immersion in 10 complete spinal cord-injured patients, 10 healthy humans before and after lower thigh capsaicin C-fiber cutaneous conduction blockade, and 10 warm-immersed healthy participants, we speculate the existence of a cutaneous pathway involving peripheral C-fiber cross-talk, partially bypassing the cord. Much remains to be learned about this unique pathway connecting the foot to ipsilateral facial skin, partially bypassing the spinal cord. However, even at this early stage of investigation, this pathway may explain previously ill-understood neurological phenomena, and, if verified by others, there is the possibility that it may be applied to helping spinal injured patients to walk again.

## Ethics Statement

This study was performed in strict accordance with the recommendations of the National Health and Medical Research Council statement on Ethical Conduct in Human Research. All procedures were approved by the Institutional Human Research Ethics Committees of Austin Health (Approval No. H2013/05045), Curtin University (Approval Nos. SMEC-60-09 and RDSE-26-15), and Murdoch University (Approval No. 2018/159). All participants gave written informed consent in accordance with the Declaration of Helsinki.

## Author Contributions

MS and MG: conceptualization, formal analysis, supervision, funding acquisition, investigation, visualization, methodology, writing-original draft, project administration, writing-review, and editing. AN: conceptualization, formal analysis, supervision, investigation, visualization, methodology, writing-original draft, writing-review, and editing. PD: conceptualization, formal analysis, investigation, visualization, methodology, writing-original draft, writing-review, and editing. DW: formal analysis, investigation, methodology, writing-original draft, writing-review and editing. JA and MM: methodology, writing-original draft, writing-review, and editing.

### Conflict of Interest Statement

The authors declare that the research was conducted in the absence of any commercial or financial relationships that could be construed as a potential conflict of interest.

## Supplementary Material

Supplementary Video 1 Cutaneous pathway for ipsilateral facial vasodilation following ice water foot immersion. The cold pressor stimulus to the foot results in proximal afferent C-fiber sensory transmission both superficially, via a cutaneous cross-talk pathway, resulting in ipsilateral facial vasodilation and, deeper nerves (tibial and peroneal) to the spinal cord with further propagation to the contralateral cerebral cortex, where it is perceived.

Supplementary Video 2 Capsaicin blocks the ipsilateral cutaneous pathway, enabling contralateral facial vasodilation. Ipsilateral lower thigh capsaicin application blocks the cutaneous cross-talk, but has no effect on sensory transmission via deeper nerves to the contralateral spinal cord. Antidromic conduction via facial C-fibers results in facial vasodilation on the opposite side to the cold pressor stimulus.

Supplementary Video 3 Ipsilateral cutaneous pathway remains intact in spinal cord injured patients. While contralateral sensory transmission is blocked by complete spinal cord injury, the ipsilateral cutaneous pathway, and resulting facial vasodilation, is unaffected.
